# “Time Slows Down Whenever You Are Around” for Women but Not for Men

**DOI:** 10.3389/fpsyg.2021.641729

**Published:** 2021-04-06

**Authors:** Joana Arantes, Margarida Pinho, John Wearden, Pedro Barbas Albuquerque

**Affiliations:** ^1^School of Psychology, University of Minho, Braga, Portugal; ^2^School of Psychology, Keele University, Keele, United Kingdom

**Keywords:** speed dating, attraction, physical attractiveness, time perception, timing

## Abstract

What happens when we unexpectedly see an attractive potential partner? Previous studies in laboratory settings suggest that the visualization of attractive and unattractive photographs influences the perception of time. The major aim of this research is to study time perception and attraction in a realistic social scenario, by investigating if changes in subjective time measured during a speed dating are associated with attraction. The duration of the dates was variable and participants had to estimate the time that passed. Among other measures, participants also rated the potential partners in terms of their physical attractiveness before and after the dates and reported if they would like to exchange contact with them. Results showed that, in a real speed dating situation, when there is a perception of the partner as being physically more attractive, women tend to overestimate the duration of that meeting, whereas men tend to underestimate its duration. Such changes may reflect evolutionary adaptations which make the human cognitive system more responsive in situations related to reproductive fitness.

## Introduction

The development of research in romantic attraction had its apogee in the 1960s and 1970s (Finkel et al., 2007) and most studies that investigated this theme demonstrated principles of attraction in laboratory settings (e.g., Walster, 1965; Byrne et al., 1967; Kephart, 1967; Stroebe et al., 1971; Dion and Dion, 1973; Pazhoohi et al., 2020). However, most of these studies were conducted using participants who have never interacted with the target of their attraction and who did not actually have the opportunity to form a real intimate relationship after the study. Finkel et al. (2007) suggested that the best way to overcome the limitations of this type of research was to study initial romantic attraction and early relationship development when the two partners meet for the first time. Romantic attraction is a complex and multidimensional construct (Gerlach and Reinhard, 2018). Typically, it refers to positive reactions that can be divided into four components: cognitive (positive thoughts and beliefs), affective (positive feelings and emotions), motivational (a desire to approach the other), and behavioral (standing or sitting closer) (Wurst and Back, 2018). Cross-cultural studies on attraction have demonstrated that the bases of relationships are not random and are strongly linked to basic mechanisms of attraction (Eastwick, 2013; Zarubko et al., 2016; Karandashev et al., 2020). Mechanisms of attraction are important because of the impact that attraction processes have on individual’s life, and because increasing knowledge about these mechanisms improves our understanding of ongoing relationship dynamics (Simpson, 1990; Finkel et al., 2007; Schindler et al., 2010). As there are methodological problems involved with retrospective reports, like systematic memory and selection biases, a real social scenario seems to be a better way to study the genesis of romantic attraction (Sprecher et al., 2008) and early relationship development from the time before two partners meet (Finkel et al., 2007; Croes et al., 2020). One such more realistic method that provides a naturalistic context to observe how prospective partners interact and that has been used in recent years by social scientists is speed dating (Fisman et al., 2006; Turowetz and Hollander, 2012; Van der Meij et al., 2019; Chang et al., 2021).

### The Speed Dating Methodology

The speed dating was invented by Rabbi Yaacov Deyo in the 90’s, with the purpose of helping single Jews of Los Angeles to meet each other. In this paradigm, people interested in meeting potential romantic partners have, approximately, 10–25 brief meetings with a series of partners (Fisman et al., 2006; Finkel and Eastwick, 2008), which typically last from 3 to 10 min each (Todd et al., 2007; Turowetz and Hollander, 2012; Ranganath et al., 2013; Janz et al., 2015). After the event, the participants report whether or not they are interested in exchanging contact with each potential partner (Finkel and Eastwick, 2008). Speed dating quickly became an element of pop culture, spread to metropolitan areas of the United States, United Kingdom, and Australia and emerged in different countries, such as Japan and South Africa (Eastwick and Finkel, 2008a). The spread of speed dating in TV programs such as “Sex and the city” (Star et al., 2000), “House M. D.” (Egan and Bookstaver, 2010), “Lost girl” (Lovretta and Fox, 2010), “Partners in crime” (Eriksen et al., 2016), and movies like “The 40-year-old virgin” (Apatow et al., 2005), “Hitch” (Smith et al., 2005), “Speed dating” (Byrd et al., 2010), “Movie 43” (Wessler et al., 2013), “The Angry Birds Movie 2” (2019) allowed speed dating to quickly evolve into a business that involves millions of people and tens of millions of dollars to access these events.

Speed dating enables researchers to access a large battery of background information about individuals before they meet one another, to introduce them to one another and to follow them after the event in order to examine relationship dynamics over the ensuing days, weeks, and beyond (Finkel et al., 2007). This methodology also allows researchers to study the dyad as the unit of analysis rather than only one person’s perspective, and to observe the attraction dynamics between two individuals who can actually create a relationship in the future (Finkel and Eastwick, 2008). Eastwick and Finkel (2008b) presented eight features of the ideal paradigm of speed dating: study real relationships with a potential future; study the interactions of both individuals; maintain experimental control; give participants multiple romantic options; get background characteristics before participants meet; implement experimental manipulations; collect “objective” ratings of participants; and follow potential relationships into the future.

In recent years, speed dating methodology has been used by different researchers (Berrios et al., 2015; Schroder-Abé et al., 2016; Nikitin et al., 2019; Van der Meij et al., 2019; Croes et al., 2020) to study several relevant aspects of research on intimate relationships. Among others, research using the speed dating methodology has studied variables such as physical attractiveness (Eastwick and Finkel, 2008c; Luo and Zhang, 2009; Back et al., 2011a), attachment (Eastwick and Finkel, 2008b; McClure et al., 2010; Spielmann et al., 2013; McClure and Lydon, 2014), personality (Luo and Zhang, 2009; Back et al., 2011b), eye-contact (Croes et al., 2020), hormones (Van der Meij et al., 2019), and sex (Eastwick and Finkel, 2008c).

### Physical Attractiveness

Physical attractiveness is related to falling in love quickly (Sangrador and Yela, 2000), and researchers have studied what makes bodies (Pazhoohi et al., 2019, 2020) and faces attractive (Thornhill and Gangestad, 1999; Little et al., 2011; Orghian and Hidalgo, 2020). In light of evolutionary psychology, research suggests that physical attractiveness is a large indicator of good health, high reproductive value and good genes. Physical attractiveness is a cue to female fertility (Buss, 1989; Miller, 2000) which seems very important in both short-term and long-term relationships. Women preferentially desire, as short-term mates, men who possess cues to good genes, but value social stability and economic security above traits relating to fertility and physical appearance for long-term relationships (Regan, 1998; Li and Kenrick, 2006). Physical attractiveness is one of the most relevant variables studied in speed dating context (e.g., Todd et al., 2007; Eastwick and Finkel, 2008a; Luo and Zhang, 2009; Bhargava and Fisman, 2014; Valentine et al., 2014; Janz et al., 2015; Jauk et al., 2016). Studies using the speed dating methodology have also shown that physical attractiveness is a very important factor in attraction for both men and women (Eastwick and Finkel, 2008c; Luo and Zhang, 2009; Back et al., 2011a; Clarkson et al., 2020; Stower et al., 2020). Luo and Zhang (2009) analyzed many variables to try to find out which of them were related to perceived attractiveness, including demographics, interests, values, political attitudes, personality, affectivity, attachment and self-esteem, finding that physical attractiveness was the most important variable in determining attraction in both sexes.

Some studies found that when people perceive a conversation partner as physically attractive, they tend to form a positive first impression (Dong and Wyer, 2014). There is much evidence that the sight of a physically-attractive person engages the appetitive motivational system, resulting in physiological responses associated with positive affect (Hughes et al., 2020). Dong and Wyer (2014) suggested that social and motivational factors that influence people’s focus of attention can have an impact on both their perceptions of duration and the judgments they base on these perceptions. Maner et al. (2007) suggested that motivational states can affect perceptual and evaluative processing of goal-relevant stimuli in a rapid and automatic manner. Despite some authors suggesting that implicit cognitive processes may be involved in mating and that cognitive resources might be attuned to stimuli related to mating opportunities (Maner et al., 2003; Silva et al., 2016), less is known about the role of automatic and instinctive cognitive processes in attraction, such as time judgments. Moreover, there are no studies using speed dating which attempt to understand what happens when people meet a physically attractive potential partner in terms of their time perception. The only five studies (Arantes et al., 2013; Odgen, 2013; Dong and Wyer, 2014; Tomas and Španić, 2016; Tian et al., 2019) that investigated the effect of attractiveness on time perception were conducted in laboratory environments, so it seems important and relevant to study this in a real scenario, such as a speed dating event. Studying time perception in these settings is important because, as mentioned previously, physical attractiveness is a crucial variable in attraction, and seems to influence temporal perception when people see a person of the opposite sex.

### Time Perception

Humans, like other animals, can estimate time (Fergunson and Martin, 1983; Block, 1990; Wearden, 2015). This ability is very important in terms of survival and, on humans, time estimation is always regarded as a part of life necessary to carry out everyday chores (Pande and Pati, 2010; Droit-Volet et al., 2015). However, subjective duration often diverges from objective duration and when this occurs time feels distorted (Sackett et al., 2010). Humans may perceive the time as passing more quickly or more slowly than the reality (Ogden et al., 2011; Wearden et al., 2017).

There are some factors studied involved in time perception accuracy, such as emotion (Droit-Volet and Meck, 2007; Droit-Volet et al., 2011; Zhang et al., 2017), arousal (Schwarz et al., 2013), alcohol (Ogden et al., 2011) attention (Gibbon et al., 1984; Zakay and Block, 1996; Brown, 2008) and memory (Brown, 1997; Staddon, 2005). An important variable that determines duration estimates is the cognitive load, that is, how cognitively demanding a task is (Block et al., 2010; Guo et al., 2019). More specifically, research has shown that the more demanding the task is (for example, when a task requires the individual to pay attention to several things during the interval to be timed), the longer the perceived duration (Khan et al., 2006; Hamamouche et al., 2018; Boris et al., 2021). This result can be explained by memory models, which state that the amount of information – or contextual changes – encoded from one interval is used for making inferences about the elapsed time during that interval (Ornstein, 1969; Block and Reed, 1978; Block et al., 2010). The effect of sex on time perception has shown conflicting results over the years (MacDougall, 1904; Block et al., 2000; Espinosa-Fernández et al., 2003; Tian et al., 2019). Nevertheless, most studies have showed that women tend to underestimate time estimations compared to men, suggesting that females may perceive time to be passing by more slowly than males (Hancock and Rausch, 2010; Glicksohn and Hadad, 2012).

Recent studies have investigated the effect of attractiveness on time perception (Arantes et al., 2013; Odgen, 2013; Dong and Wyer, 2014; Tomas and Španić, 2016; Tian et al., 2019). Odgen (2013) conducted a study with female participants exploring whether the attractiveness of a female face – presented for a short interval of time – affected the estimated duration of that stimulus. Results showed that participants judged female unattractive faces to last for a shorter time than neutral and attractive faces of the same duration. On the other hand, Arantes et al. (2013) tested the hypothesis that a female’s duration estimates of briefly-viewed male, but not female, photos would be modulated by attractiveness. Their results showed that duration estimates of attractive male photos were significantly longer than corresponding estimates for attractive and unattractive female photos. Tomas and Španić (2016) asked female participants to perform a temporal bisection task using female faces stimuli differing in facial expression (angry or neutral) and attractiveness (attractive or unattractive). They found that participants overestimated the angry faces’ durations compared to the neutral faces, but only for the attractive face condition. Findings from these three studies are consistent with the hypothesis that the timing system contains adaptations which provide flexibility in situations related to reproductive fitness. However, these authors have conducted their studies only with a sample of female participants, and thus sex comparisons were not possible.

Dong and Wyer (2014) conducted an experiment in which participants engaged in an 8-min conversation over the internet with an opposite sex person in which they could only hear each other’s voices without a visual display. Before the conversation, each participant received an attractive or unattractive photo that they thought was of the potential partner, but which was in fact manipulated by the researcher. The authors concluded that when both male and female participants perceived a partner as physically attractive, immediately after the conversation they estimated that the time engaged has been passed quickly because they based their judgment on the degrees of involvement. Tian et al. (2019) aimed to investigate whether sex modulates the effect of attractiveness on time perception, suggesting that sex seems to have an important role. In particular, they found that, for both sexes, the duration estimates of attractive opposite-sex faces were longer than for unattractive opposite-sex faces, but that women perceived the duration of attractive same-sex faces as being longer than for unattractive same-sex faces.

However, these experiments were conducted in a laboratory setting, and important cues of attractiveness, such as eye contact, smiling and body language (Muehlenhard et al., 1986) were not present. Therefore, it seems important to study the relationship between attractiveness and time perception in a conversation in a more naturalistic scenario. In addition, many questions remain unanswered: Will similar results be obtained in a realistic scenario, such as a speed dating? Will men and women allocate different mental resources to evaluate characteristics of the potential partners that are important to them, and thus duration estimates will be different when they fell attracted to their potential partners?

### Current Study

The major aim of the present study is to investigate the potential relationship of physical attractiveness and temporal perception in a relationship initiation using a speed dating methodology. Our first hypothesis is that temporal perception in a speed dating event is related to the attraction that the participant feels toward the potential partner (Arantes et al., 2013). However, we expect this relationship to be different for males and females. More specifically, we hypothesize that: (i) For women, when they perceive their potential partner as very attractive, they will estimate the duration of the date as being longer; and (ii) For men, when they perceive their potential partner as very attractive, they will estimate the duration of the date as being shorter.

These hypotheses can be understood in the light of the evolutionary psychology (Buss, 1995). More specifically, our first hypothesis is based on Trivers (1972) parental investment theory, which assumes that women are the more investing sex. Therefore, when a woman perceives a potential partner as physically attractive she pays attention to several other characteristics of that man in order to make a reasoned choice (e.g., economic resources and intelligence), allocating many mental resources in that assessment. The use of these cognitive resources would make the perceived duration of the date longer (Ornstein, 1969). For men, we derived the opposite hypothesis, because according to the parental investment theory, males tend to be less selective than women and they may feel attracted to potential partners based mostly on their physical attractiveness (Trivers, 1972; Todd et al., 2007; Bokek-Cohen et al., 2008). So, when men have a meeting with a potential partner they perceived as physically attractive, they do not use a lot of resources evaluating other characteristics of the partner and they may feel more motivated to talk and experience this conversation as being enjoyable. Consequently, they may estimate the time that passed as being shorter. This hypothesis is also based on the idea that “time flies when you are having fun,” supported by prior studies (e.g., Danckert and Allman, 2005; Glabe and Poole, 2012) that showed time is underestimated when participants are interested and motivated.

The secondary aim of our study is to analyze the influence of meeting a potential partner on an attractiveness judgment. We hypothesize that in a speed dating context, when participants stay interested and want to exchange contacts with a potential partner to keep in contact in future, the perceived attractiveness will increase, and when they do not want it, it will not change. Many studies showed that knowing characteristics of a person changes the perception of attractiveness (Tartaglia and Rollero, 2015; Gerlach and Reinhard, 2018). For example, both laboratory (e.g., Lewandowski et al., 2007) and naturalistic (e.g., Kniffin and Wilson, 2004) experiments show that personality and other non-physical characteristics affect physical attractiveness judgments. Nevertheless, there is no research using speed dating to understand the effect of characteristics of the potential partner by asking the participant to rate the physical attractiveness of the partner before and after the meeting. In addition, there are no studies in which participants rate the potential partner in terms of physical attractiveness before and after the speed dates. In the majority of studies using real contexts such as speed dating, physical attractiveness was measured by external observers (e.g., Back et al., 2011a; Jauk et al., 2016) and in the few studies in which attractiveness of potential partner was measured by participants, this evaluation is made only at one time in the event (e.g., Selterman et al., 2005), so it could be influenced by other characteristics of the partner and by whether they liked them or not.

## Materials and Methods

### Participants

Our sample was composed of 37 participants, 18 females and 19 males. More specifically, in the first speed dating session there were 21 participants, of which 11 were females and 10 males, and in the second event 16 participants, 7 females and 9 males. This yielded a total of 173 speed dates. Participants were aged between 18 and 27 years old (*M* = 21.78, *SD* = 2.36). Males (*M* = 22.19; *SD* = 2.33) were older than females (*M* = 20.88; *SD* = 2.08), *t*(250.20) = −4.40, *p* < 0.001. None was involved in a romantic relationship. Males reported having more relationships in the past (*M* = 3.30; *SD* = 2.95) than females (*M* = 2.13; *SD* = 1.67), *t*(250.20) = −4.40, *p* < 0.001.

Volunteers were recruited through online social networks and advertisements (i.e., flyers) at Minho University and at the bar where the event was held. More specifically, participants were invited to participate in a speed dating session that would occur in a bar at a specific date, with limited space available. It was also said that the event was free but required registration (though email). Finally, it was mentioned that participants needed to be between 18 and 27 years-old, could not be in any kind of romantic relationship, and needed to be heterosexuals. Participants did not receive any kind of reward for participation besides the snack we offered after the event (and that was not advertised), while the participants were waiting for the remaining participants to arrive.

### Measures

#### Demographic Questionnaire

Participants answered to a demographic questionnaire that included questions about their age, sex, nationality, and number of previous romantic relationships.

#### Pre-event Questionnaire

Before the event, we presented photographs of all potential partners and participants rated them in terms of their physical attractiveness, using a 10-point scale, from 1 (“not attractive at all”) to 10 (“very attractive”).

#### Post-meeting Questionnaire

After each date, participants answered to a brief questionnaire in which they were asked to evaluate: how long the date lasted (by marking on a line from 1 to 8 min); the physical attractiveness of the partner, on a 10-point scale, from 1 (“not attractive at all”) to 10 (“very attractive”); how much attraction they felt toward the potential partner; and how much attraction they believed the partner had toward them, on a 10-point scale, from 1 (“nothing”) to 10 (“a lot”). Then, they were asked whether they would like to exchange contact details with that person. Finally, participants were asked if they already knew that person. Those that answered affirmatively were asked to specify the degree of proximity.

### Equipment

To take the photographs of participants before the event we used an instant camera *Fujifilm instax wide.* Using this camera enabled us to take a photo just before the event in a more informal and comfortable way, because we could give participants their own photos at the end of the event.

### Procedure

The experiment was approved by the ethical committee of University of Minho and was conducted in accordance with their guidelines. As instructed on the advertisements, participants interested in participating on the speed dating events sent an e-mail to the researchers. Later, they were contacted and given a brief explanation about the experience and some information about the event.

The event was held in a bar near to Minho University and before the event males and females were directed to different spaces of the bar: men to the right and women to the left. Those spaces were separated by a wall and participants entered by different doors to avoid visual contact with the other sex participants. Then, all participants received a sticker with a number, and were asked to take individual photos. They were offered a snack while they waited. Afterward, each participant was given photos of the potential mates, printed on paper, and asked to evaluate them in terms of physical attractiveness.

In each speed dating event, participants experienced dates with all the opposite sex participants, and each date varied in duration from 180 to 375 s (*M* = 269 s; *SD* = 73.12 s; nine durations: 180, 205, 240, 265, 300, 325, 350, 360, and 375 s) and the durations of the dates were selected in randomized order. Immediately before the event, the participants were asked to remove their watches and cell phones. Each date ended with the sound of a bell, followed by a quick post-meeting survey that was given to every participant about the concluded date. After each interaction men moved to the next date and women remained in the same table. At the end of all the speed dates, participants answered a demographic questionnaire. After the event, those who indicated mutual interest received each other’s contact details via email.

Follow-up sessions 3 and 6 months later were held to know if any participants got involved in a romantic relationship.

### Data Analysis

The data collected in this research were analyzed with Statistical Package for Social Sciences (SPSS; v. 24). Analysis involved *t*-tests for dependent samples to analyze differences between subjective and real time, Pearson’s correlations to study associations between time perception and attractiveness measures. We have also done 2 × 2 × 2 repeated-measures analysis of variance (ANOVA) with male/female and exchange/not exchange contact as between-subjects and physical attractiveness before/after the date as within-subject variables (Field, 2005), and regression analyses. To analyze the data, participants that had already met the speed date partner were excluded.

## Results

**FIGURE 1:**
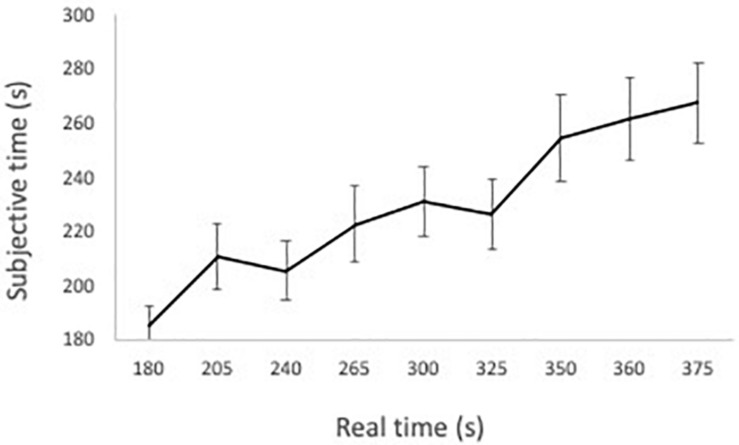
Average estimated durations across real time.

In general, participants tended to estimate the duration of the date as being shorter (*M*_subjective_ = 221.96 s, *SD* = 81.19 s) than it was in reality (*M*_reality_ = 268.26 s, *SD* = 72.76 s), *t*(320) = 9.39, *p* < 0.001. Figure 1 shows that as actual time increases, the perceived time also increases. This shows that participants were able to estimate the time.

**FIGURE 2:**
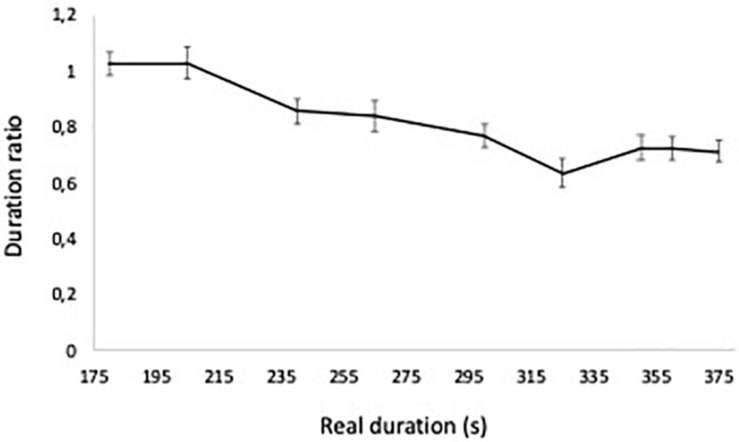
Ratio of estimated duration to real duration, across real time.

To measure timing, we calculated the ratio between subjective duration and real duration of the date. Figure 2 presents the ratio of subjective/real time across real time and shows that participants overestimated the time for durations below 205 s and underestimated the durations above that value. This pattern shows that the estimated time in this research is according to Vierordt’s Law, von Vierordt (1868), that says that for shorter durations participants tend to overestimate the time and for longer durations underestimate it (Lejeune and Wearden, 2009).

### Attractiveness and Timing – Zero Order Correlations

Tables 1, 2 show the Pearson’s correlations of associations between time perception, perceived attractiveness before and after the date, attraction felt toward the partner, and expected attraction toward them – for females and males, respectively. Table 1 shows that the more female participants perceived a potential partner to be physically attractive before and after the meeting, the more they reported attraction to the partner, *r*_before_(149) = 0.57, *p* < 0.001, *r*_after_(163) = 0.89, *p* < 0.001. Similarly, Table 2 show that the more males perceived a potential partner as being physically attractive before and after the date, the more attraction to the potential partner they reported, *r*_before_(160) = 0.54, *p* < 0.001, *r*_after_(160) = 0.86, *p* < 0.001. In addition, the more attracted the participants felt to their partners, the more attracted they judged that the partner would be to them. This was observed in both females and males, *r*_female_(163) = 0.63, *p* < 0.001, *r*_male_(163) = 0.74, *p* < 0.001. Results also showed that the higher the perceived attractiveness of the partner before the date, the higher the perceived attractiveness of the partner after the date, for both sexes, *r*_female_(149) = 0.61, *p* < 0.001, *r*_male_(163) = 0.57, *p* < 0.001. Furthermore, the higher the partners’ expected attraction toward them, the higher the perceived attractiveness before and after the date, for females, *r*_before_(163) = 0.32, *p* < 0.001, *r*_after_(163) = 0.55, *p* < 0.001, and for males, *r*_before_(162) = 0.40, *p* < 0.001, *r*_after_(163) = 0.56, *p* < 0.001.

**TABLE 1:** Correlations for duration ratio, physical attractiveness before the date, physical attractiveness after the date, participant attraction toward partner and perception of partner attraction toward her, for females.

	Duration ratio	PA before	PA after	Attraction toward partner	Expected attraction toward them
Duration ratio					
PA before	0.26**				
PA after	0.19*	0.61***			
Attraction toward partner	0.24**	0.57***	0.89***		
Expected attraction toward them	0.06	0.32***	0.55***	0.63***	

PA, physical attractiveness. *p < 0.05, **p < 0.01, ***p < 0.001.

**TABLE 2:** Correlations for duration ratio, physical attractiveness before the date, physical attractiveness after the date, participant attraction toward partner and perception of partner attraction toward him, for males.

	Duration ratio	PA before	PA after	Attraction toward Partner	Expected attraction toward them
Duration ratio					
PA before	−0.18*				
PA after	−0.20*	0.57***			
Attraction toward partner	−0.23**	0.54***	0.86***		
Expected attraction toward them	−0.18*	0.40***	0.56***	0.74***	

PA, physical attractiveness. *p < 0.05, **p < 0.01, ***p < 0.001.

Regarding time perception, the more females judged a potential partner as physically attractive before and after the date, and the more attracted they were to him, the longer they estimated the duration of the speed date, *r*_before_(148) = 0.26, *p* < 0.01, *r*_after_(162) = 0.19, *p* < 0.05, *r*_attraction_(162) = 0.24, *p* < 0.01. However, in case of males, the greater the judgments of the partner as physically attractive before and after the meeting and the more attraction they felt to their female potential partners, the shorter they estimated the duration of the speed date, *r*_before_(159) = −0.18, *p* < 0.05, *r*_after_(159) = −0.20, *p* < 0.05, *r*_attraction_(159) = −0.23, *p* < 0.01. Results also showed that the higher the women expected attraction toward them, the shorter men estimated the duration of the speed date, *r*_before_(161) = −0.18, *p* < 0.05.

We then compared the correlation coefficients related to time perception of both men and women, by transforming the correlation coefficient values into *z* scores and using the following formula: *Z*_observed_ = (*z*_women_ − *z*_men_)/(square root of [(1/*N*_women_ − 3) + (1/*N*_men_ − 3)], in which N corresponds to the sample size. Results showed that there were no differences in the magnitude of the correlations coefficients of both men and women, with all *ps* > 0.05 (see Table 3).

**TABLE 3:** Comparison of the correlation coefficients related to time perception of both men and women.

Variables correlated with duration ratio	*r* women	*r* men	*Z* women	*Z* men	*N* women	*N* men	*Z* observed	*p*
PA before	0.26	−0.18	0.2661	0.1820	148	159	0.7291	0.2327
PA after	0.19	−0.20	0.1923	0.2027	162	159	−0.0923	0.4641
Attraction toward partner	0.24	−0.23	0.2448	0.2342	162	159	0.0941	0.4641
Expected attraction toward them	0.06	−0.18	0.0601	0.1820	162	158	−1.0799	0.1401

### Attractiveness and Timing – Regression Analyses

The zero-order correlations presented above demonstrated that, for both males and females, the perceived date duration was significantly correlated with the perception of the partner’s physical attractiveness before and after the date, and the attraction felt toward the partner. The perceived date duration was also associated with the expected attraction the partners felt toward them, but only for men. However, because these variables were low-to-moderately intercorrelated, it was of interest to determine their explanatory power. We therefore performed stepwise multiple regression analyses – separated for each sex –, with perceptions of date durations as dependent variable the four variables and were each regressed onto the four predictor variables mentioned above. These analyses are presented in Table 4. Results indicated that the regression model was statistically significant for men, *F*(1,145) = 6.187, *p* < 0.05, and women, *F*(1,160) = 9.967, *p* < 0.01. However, the models were weak in explanatory power, accounting only for approximately 4% (*R*^2^) of the total variance in men’s perceptions of date duration and 6% (*R*^2^) in women’s perceptions of date duration. In addition, the model excluded, for both sexes, three variables, namely perception of the partner’s physical attractiveness before the date, perception of the partner’s physical attractiveness after the date, and expected attraction the partners felt toward them. Therefore, these results indicated that, among the four variables examined, the attraction felt toward the partner was the strongest and unique predictor of both men’s and women’s perceptions of date duration.

**TABLE 4:** Multiple regression models predicting perceptions of date duration.

	Variable	β	*SD*	β	*t*	*P*
Model 1 (women)	Constant	0.631	0.069		9.118	0.000
	Attraction toward partner	0.040	0.013	0.242	3.157	0.002
Model 2 (men)	Constant	1.053	0.070		14.976	0.000
	Attraction toward partner	–0.031	0.012	–0.201	–2.468	0.015

### Perception of Physical Attractiveness of the Partner

Data were entered into a 2 × 2 × 2 repeated-measures analysis of variance (ANOVA) with male/female and exchange/not exchange contact as between-subjects and physical attractiveness before/after the date as within-subject variables (Table 5). This analysis found a significant main effect of attractiveness before and after the date, *F*(1,305) = 21.38, *p* < 0.001. *Post hoc* analysis determined that participants tend to evaluate the physical attractiveness of the partner as being higher after the date (*M* = 5.74) compared with before the date (*M* = 5.26), *p* < 0.05. Results also showed a significant interaction between physical attractiveness before/after and sex of participant, *F*(1,305) = 13.39, *p* < 0.001. *Post hoc* analysis showed that women rated the partners as being more physically attractive after the date (*M* = 5.64) compared to before the date (*M* = 4.77), *p* < 0.05. Men’s perceptions of their partners’ attractiveness were similar before (*M* = 5.71) and after the date (*M* = 5.83), *p* > 0.05. There was a significant interaction between exchange/not exchange contact and physical attractiveness before/after, *F*(1,305) = 29.56, *p* < 0.001. When participants chose to exchange contacts with a partner, their perception of their partner’s physical attractiveness increased after the date (*M* = 6.82) compared with the rating before the date (*M* = 5.85), *p* < 0.05. On the other hand, when participants did not show interest in exchanging contacts, the physical attractiveness rating of the potential partner did not change after the date (*M* = 4.44) compared with before the date (*M* = 4.55), *p* > 0.05. The three-way interaction between sex, contact and physical attractiveness was not statistically significant, *F*(1,305) = 0.02, *p* = 0.893.

**TABLE 5:** Physical attractiveness of the partner perceived by the participant before and after the speed dates in function of the interest in exchange or not contact with them for females and males.

		Contact	No contact
**Physical attractiveness before**	Female	5.41 (2.02)	3.91 (1.87)
	Male	6.3 (1.50)	5.08 (1.47)
	Total	5.85 (1.84)	4.55 (1.76)
**Physical attractiveness after**	Female	6.71 (1.50)	4.19 (1.72)
	Male	6.93 (1.41)	4.64 (1.70)
	Total	6.82 (1.46)	4.44 (1.72)

*The values presented are mean and standard deviation.*

### Dyadic Analyses

In order to conducted the dyadic analyses, we paired each female with each male speed-dating partner. Table 6 shows the Pearson’s correlations between females’ and males’ time perception, perceived attractiveness before and after each date, and attraction felt toward each potential partner. Results showed that there was a significant positive correlation between each dyadic duration ratio, *r*(161) = 247, *p* < 0.05, suggesting that the longer a woman perceived the duration of a date, the longer the man also perceived that date duration. Results also showed a positive significant correlation between women’s perceptions of physical attractiveness of the partner before the date and men’s expected attraction toward them, *r*(148) = 0.187, *p* < 0.05, indicating that the higher the women perceived the physical attractiveness of the partner before the date, the more their men believed women were attracted to them. In addition, our data showed a negative significant correlation between women’s perceptions of physical attractiveness after the date and men’s perception of their physical attractiveness before the date, *r*(162) = −0.180, *p* < 0.05, suggesting that women that assessed their partner as having higher levels of attractiveness after the date tended to be evaluated by those partners as having low levels of attractiveness before the date. There was also a negative significant correlation between women’s’ expected attraction from their partner toward them and men’s perception of women physical attractiveness before the date, *r*(162) = −0.180, *p* < 0.05, suggesting that women that believed their partners were attracted toward them tended to be evaluated with low levels of attractiveness by their partners before the date. Finally, results showed that there was a negative significant correlation between women’s duration ratio and men’s expected attraction from their partner toward them, *r*(162) = −0.195, *p* < 0.05, suggesting that the longer women perceived the duration of a date, the less their partners expected them to be attracted toward them.

**TABLE 6:** Correlations between females and males time perception, perceived attractiveness before and after each date, and attraction felt toward each potential partner.

	Men’s duration ratio	Men’s PA before	Men’s PA after	Men’s attraction toward partner	Men’s expected attraction toward them
Women’s duration ratio	0.247***	0.017	–0.019	–0.115	−0.195*
Women’s PA before	–0.091	0.111	0.116	0.139	0.187*
Women’s PA after	–0.014	−0.180*	–0.102	–0.097	0.040
Women’s attraction toward partner	–0.085	−0.173*	–0.072	–0.031	0.053
Women’s expected attraction toward them	–0.054	–0.079	0.005	–0.035	–0.035

PA, physical attractiveness. *p < 0.05, **p < 0.01, ***p < 0.001.

Out of the 173 speed dates, there were 32 matching (18.50%), that is, reports of mutual interest and consequently exchanges of contact details. For follow-up, we contacted all participants. Participants were contacted after 3 and 6 months, and of those who responded, we were informed that three intimate relationships were formed.

## Discussion

The main objective of this research was to study time perception and attraction in a realistic social scenario by investigating if changes in subjective time measured during a speed dating session were related with attraction. The duration of the dates was variable and participants had to estimate the time that passed. Participants were asked to rate potential partners in terms of their physical attractiveness before and after the dates and to report if they wanted to exchange contacts with them. Our data suggest, consistently with our hypotheses, that the estimated time of the dates were associated with the physical attractiveness of the potential partners perceived by participants.

More specifically, our results showed that the more females rated a potential partner as physically attractive, the longer they perceived the duration of the date. That goes along with the popular idea that “time slows down whenever you are around” (Swift, 2010). This may be due to a bigger allocation of women’s cognitive resources to process more information of the meeting (Loftus et al., 1987) and of the potential partner they are interested in. More specifically, even though physical attractiveness is important in a potential partner, for women there are other characteristics that may have a higher value, such as good economic prospects (Buss and Barnes, 1986; Bech-Sørensen and Pollet, 2016). Therefore, searching for cues of positive traits in a potential mate requires the use of cognitive resources. Besides that, research has shown that when women perceive the partner as attractive, they tend to be more motivated to make a good impression on the partner and pay more attention to the things they say that might influence this impression (Dong and Wyer, 2014). According to Ornstein’s storage size model (Ornstein, 1969; Sasaki and Yamada, 2017), when people store more information in memory, they tend to perceive the duration of that interval of time as being longer. Furthermore, women may consider the experience with a partner who they consider physically attractive as positive in an emotional way. This result is also consistent with that study of Kellaris and Kent (1992) in which time did seems to slow downs when participants were exposed to positively valenced music, compared to participants exposed to negatively valenced music. The authors suggested that when people receive positive emotional information they tend to invest more cognitive resources in listening to music. Therefore, they tend to perceive the received stimulus information as larger and remember the event as being longer. Besides that, a study conducted by Zhang et al. (2017) showed a reliable sex differences in temporal distortion with an emotional stimulus. Women, compared to men, tended to overestimate the durations of emotional words.

However, for men, our results showed that time does not seems to slow down whenever someone attractive is around. In fact, the more males rated a female participant as physically attractive, the shorter they perceived the duration of the speed date. This seems to be consistent with the idea that “time flies when you are having fun.” Research has shown that men’s preferences for potential mates are based mostly in physical attractiveness (Lippa, 2007; Todd et al., 2007; Eastwick et al., 2011). Therefore, when they have a meeting with a potential partner that they perceive as being physically attractive, they do not need to spend much cognitive resources searching for other cues, feeling automatically motivated to be with her. Consequently, they will tend to estimate the time that passed as being shorter. This result also suggests that time perception in males during the dates may be affected by motivation because, according to previous literature, positive approach motivation causes the perception of time to be shorter (Glabe and Poole, 2012). Besides that, the subjective perception of the passage of time seems to be an important component to evaluate the experience of boredom (Danckert and Allman, 2005). So, when males are interested and motivated in the date with a physical attractive potential partner, they tend to estimate the date duration as shorter and, on the other hand, this time underestimation reinforces the perception of an interesting date (Sackett et al., 2010). Underestimation of the duration of the date may prolong approach-motivated behavior (Glabe and Poole, 2012) and this increases the probability of a successful mating. On time, Einstein said “Put your hand on a hot stove for a minute and it seems like an hour. Sit with a pretty girl for an hour, and it seems like a minute.”

According to Trivers’ (1972) theory, the relative parental investment of the sexes in their offspring is the key variable controlling the operation of sexual selection. Sexual intercourse for a male is a small investment, but for a female can produce a 9-month investment, at least. For a female, this investment requires more choosiness in the partner choice. Besides that, prior research showed that females tend to be more selective (Kurzban and Weeden, 2005) and more discriminating (Todd et al., 2007) than males. Therefore, it is expected that females allocate more attention to capturing a greater number of characteristics of the potential partner in addition to physical attractiveness, such as intelligence, personality, earning prospects and other signs suggesting he could be good partner in the future. This process seems to imply an exhaustive evaluation in the first meeting which requires the allocation of a lot of cognitive resources. On the other hand, men are attracted by fewer characteristics of the partner compared to females (Luo and Zhang, 2009). So, males do not use so much energy and resources in cognitive processing of information and focus more energy in having fun with the partners they perceived as being more attractive. Such changes may reflect evolutionary adaptations which make the human cognitive system more responsive in situations related to reproductive fitness.

Williams (2012) suggested that sex differences in timing might be due to the effects of circulating estrogen in adult females versus testosterone in adult males. Besides that, gonadal hormones had been found to influence sexual motivation (Wallen, 2001). In men, testosterone increases interest in a woman, engagement in self-presentation, smiling and making eye contact (Roney et al., 2006; Meij et al., 2011; Thornhill et al., 2013). Meij et al. (2011) suggested that during encounters with the opposite sex, testosterone may promote the display of affiliative behaviors that increase a man’s mating prospects and during social contact with a potential partner testosterone is linked to the initiation of courtship behaviors. On the other hand, in women, estradiol seems to be a significant positive predictor of sexual desire (Puts et al., 2013; Roney and Simmons, 2013).

Estradiol is one of the natural estrogens and has been shown to increase striatal dopamine release, that may modify temporal perception and timing performance in a manner similar to indirect dopamine agonists such as amphetamine and cocaine (Pleil et al., 2011). Estrogen as a dopamine agonist facilitates striatal dopaminergic activity (Sandstrom, 2007), stimulating the dopaminergic transmission and, consequently, producing an overestimation of time intervals (Cheng et al., 2006). Pleil et al. (2011) investigated sex differences in the rapid and acute effects of estradiol on time perception in adult male and female rats. According to the authors, their results are consistent to the idea that there are multiple mechanisms of estrogen’s action in the striatum that modulate dopaminergic activity and are differentially organized by gonadal steroids during early brain development. Additionally, Becker (1999) found that striatal dopaminergic release is affected by estrogen only in females. The striatum is one of the components of the basal ganglia that have been suggested to be a fundamental component of the neural basis of timing (Ivry and Spencer, 2004) and multiple studies, specifically with patients with dopamine system disorders as Parkinson (Leranth et al., 2000; Michel et al., 2002), and schizophrenia (Seeman and Lang, 1990; Riecher-Rössler and Häifner, 1993; Michel et al., 2002) and others, found an interaction between gonadal steroid hormones such as estrogen in basal ganglia mechanisms (Hartesveldt and Joyce, 1986). Therefore, because of the fact that estrogen is a predictor of sexual desire and sexual motivation, this may increase their circulation in women during a speed date with physically attractive partners and, subsequently, increase dopamine release in striatum. Besides that, some studies found that women, on average, have higher presynaptic dopamine synthesis capacity (Laakso et al., 2002) and lower D2 receptor affinity (Pohjalainen et al., 1998) that suggests an increased endogenous dopamine in women’s striatum, comparing to men. So, neural sex differences in dopaminergic circuits in the striatum could explain this sex difference on the influence of physical attractiveness in time perception. It is thus possible that sexual hormones on males have an opposite effect in striatum (Myers et al., 2003).

Our results may diverge from Dong and Wyer (2014) study because sex differences in their study could be masked by lack of cues in the interaction that could influence attraction mechanisms. Specifically, the reduction of non-verbal information may influence the response of females more than males because, according to a vast literature (Mehrabian, 1972; Mehrabian and Ksionzky, 1972; Zahn, 1973, 1975), females are more sensitive to non-verbal information and males to a verbal information.

Our study also demonstrates that for the decision of exchanging or not contact with the partner, physical attractiveness seems to be an important factor for both sexes because when participants perceived the partners as physically attractive, they tended to exchange contacts with them. In addition, consistent with our second hypothesis, the physical attractiveness of the potential partner perceived by the participant changes according to the interest in exchanging contact with him/her. In other words, interest or not in the meeting with a potential partner and the desire or not to keep in contact in the future influences their perceived physical attractiveness. Particularly, when participants are interested in a potential partner at the end of the date, they perceive their physical attractiveness as being higher compared to the initial evaluation (i.e., before the date). When participants are not attracted to partners at the end of the date, expressing the desire not to exchange contacts with them, they not change their evaluation of the potential partner’s physical attractiveness. These results suggest that there may be an effect of other characteristics of the potential partner in the evaluation of physical attractiveness. This is supported by some laboratory studies that have shown that the evaluated attractiveness of opposite-sex people is influenced by their personality. For example, Lewandowski et al. (2007) found that when a person was presented with positive personality information about the person shown in a photograph, participants rated that person as more physically attractive and when photographs were paired with negative personality information the person depicted was rated as less physically attractive. These results are also consistent with Kniffin and Wilson’s (2004) naturalistic studies that showed that non-physical characteristics such as familiarity, liking, respect, talent, and effort have a great influence on physical attraction judgments.

### Limitations and Future Research

First, previous studies found that preferences in mate selection are influenced by the type of desired relationship, short or long-term. Thus, in future research it seems relevant to question participants in the speed dating event about whether they would like to have a short or long-term relationship with the partners they show an interest in exchanging contacts with. Second, this research shows that in a realistic scenario where two people meet each other, changes occur in time perception and it seems plausible to us that other implicit cognitive processes are affected in this context. However, there are no studies about other implicit measures in speed dating events, such as memory or attention, and future research should focus on this theme. Third, in terms of time perception and attractiveness, our data were correlational, so do not provide evidence for a causal influence of physical attractiveness on timing. Our results suggest that the two variables are associated but it would be interesting to understand if there is a causal relation between them. Third, there were 32 (18.50%) exchanges of contact details but only three intimate relationships were formed and lasted at least 6 months. It would be interest to investigate in future studies which variables have contributed to the development of an intimate relationship after the speed-dating. Fifth, participants were relatively young people, which may represent a limitation of the present study. Research has shown that men tend to prefer females at the age at which fertility peaks in order to increase their reproductive success (Conroy-Beam and Buss, 2019). In future research, it seems important to understand if the results of this study are applicable to older ages, in particular in postmenopausal women. If time perception in dating situations is an adaptive mechanism for mating, this bias should no longer occur in post-reproductive, menopausal women (Cyrus et al., 2011). Finally, our results based on stepwise multiple regression analyses showed that the attraction felt toward the partner was the strongest and unique predictor of both men’s and women’s perceptions of date duration. These results support the idea that when individuals are exposed to opposite-sex persons to whom they feel an attraction, their timing system is affected – women tend to overestimate, whereas men tend to underestimate the passage of time. However, it is still important to notice the weak explanatory power of the models, which indicates that there are other contributing factors to time perception that need to be explored in future research.

### Conclusion

Results of the present study open access to new knowledge about what happens when an individual feels attracted to another of the opposite sex. This is the first research to study time perception in a real speed dating event. Our data show that changes in timing are associated with attraction, particularly when the meeting is with someone perceived as physically attractive. On one hand, the more women perceive men in a date as physically attractive, the longer they estimate the duration of the meeting. On the other hand, the more men rate the potential partner as physically attractive, the shorter they estimate the duration of the date. This research is also the first to analyze the perceived physical attractiveness of a potential partner before and after the meeting. Our results demonstrate that when people show an interest in a potential partner, the perceived physical attractiveness of the partner increases.

Our research will help to understand what happens automatically in the cognitive system in situations related to interpersonal attraction and provides new evidence for probable human timing adaptations that may respond differently according to sex to a stimulus related with mating. So, it seems that implicit cognitive processes may be involved in attraction when people meet for the first time and could be explained in the light of evolutionary psychology. Men and women have different selection pressures and thus value different characteristics in a potential partner. As women value highly other features besides physical attractiveness, in a mating situation such as a speed dating, they will allocate more cognitive resources to process more information of an attractive men – overestimating the duration of that date. As men value mostly physical attractiveness in a potential partner, when males are interested and motivated in the date with a physical attractive potential partner, they tend to estimate the date duration as shorter. In addition, our study opens a new line of research on intimate relationships outside the laboratory, in a real-life event.

## Data Availability Statement

The raw data supporting the conclusions of this article will be made available by the authors, without undue reservation.

## Ethics Statement

The studies involving human participants were reviewed and approved by Ethics Committee from the University of Minho. The participants provided their written informed consent to participate in this study.

## Author Contributions

JA was responsible for the planning of the experiment, data collection, data analysis, and writing of the manuscript. MP was involved in the data collection, data analysis, and writing of the manuscript. JW and PA were involved on the planning of the experiments and discussion of the results. All authors contributed to the article and approved the submitted version.

## Conflict of Interest

The authors declare that the research was conducted in the absence of any commercial or financial relationships that could be construed as a potential conflict of interest.
